# Inflammation and Notch signaling: a crosstalk with opposite effects on tumorigenesis

**DOI:** 10.1038/cddis.2016.408

**Published:** 2016-12-08

**Authors:** Chiara Fazio, Luigi Ricciardiello

**Affiliations:** 1 Department of Medical and Surgical Sciences, University of Bologna, Bologna, Italy

## Abstract

The Notch cascade is a fundamental and highly conserved pathway able to control cell-fate. The Notch pathway arises from the interaction of one of the Notch receptors (Notch1–4) with different types of ligands; in particular, the Notch pathway can be activated canonically (through the ligands Jagged1, Jagged2, DLL1, DLL3 or DLL4) or non-canonically (through various molecules shared by other pathways). In the context of tumor biology, the deregulation of Notch signaling is found to be crucial, but it is still not clear if the activation of this pathway exerts a tumor-promoting or a tumor suppressing function in different cancer settings. Untill now, it is well known that the inflammatory compartment is critically involved in tumor progression; however, inflammation, which occurs as a physiological response to damage, can also drive protective processes toward carcinogenesis. Therefore, the role of inflammation in cancer is still controversial and needs to be further clarified. Interestingly, recent literature reports that some of the signaling molecules modulated by the cells of the immune system also belong to or interact with the canonical and non-canonical Notch pathways, delineating a possible link between Notch activation and inflammatory environment. In this review we analyze the hypothesis that specific inflammatory conditions can control the activation of the Notch pathway in terms of biological effect, partially explaining the dichotomy of both *phenomena*. For this purpose, we detail the molecular links reported in the literature connecting inflammation and Notch signaling in different types of tumor, with a particular focus on colorectal carcinogenesis, which represents a perfect example of context-dependent interaction between malignant transformation and immune response.

## Facts

- Notch signaling is an evolutionarily conserved molecular pathway, crucial for the development and homeostasis of most tissues.
- The Notch receptors, a family of trans-membrane proteins, are also known to be involved in the pathogenesis of a spectrum of human diseases, including cancer. Nowadays, Notch receptors are reported to act both as tumor suppressors and oncogenes.
- Inflammation is characterized by a complex mixture of mediators that have a strong impact on normal and cancer cells.
- The role of Notch in inflammatory-driven tumors is now emerging, but its effect is still controversial.

## Open question

- How does inflammation influence Notch signaling?
- Is the inflammatory context a contributing factor for Notch pathway activation?
- What is the relevance of the Notch pathway in inflammatory-driven cancers?
- Can the targeting of inflammation impact Notch pathway activity?

Notch signaling is a molecular pathway used as a general developmental tool for controlling organ formation and morphogenesis in both invertebrate and vertebrate organisms,^1^ and avails itself of a direct cell-cell model of communication.^2^ The signals exchanged between neighboring cells through the Notch pathway can orchestrate a surprisingly wide spectrum of specific programs, including differentiation, proliferation and apoptosis, which are able to influence cell-fate and to regulate tissue homeostasis.^3^

Importantly, the deregulation of Notch signaling has been found involved in many pathological processes, including cancer.^4^^5^ In particular, a double role of the Notch pathway, acting as both tumor suppressor or tumor promoter, has been reported.^6^

Although the inflammatory microenvironment arises from a normal host defense with the goal of inducing pathogen elimination, it is well documented that low-grade/chronic inflammation plays a pivotal role in cancer promotion.^7^ Moreover, the tumor microenvironment, which is largely orchestrated by inflammatory cells and their secreted factors, is an indispensable participant in cellular apoptosis/survival and migration.^8^

**Figure 1:**
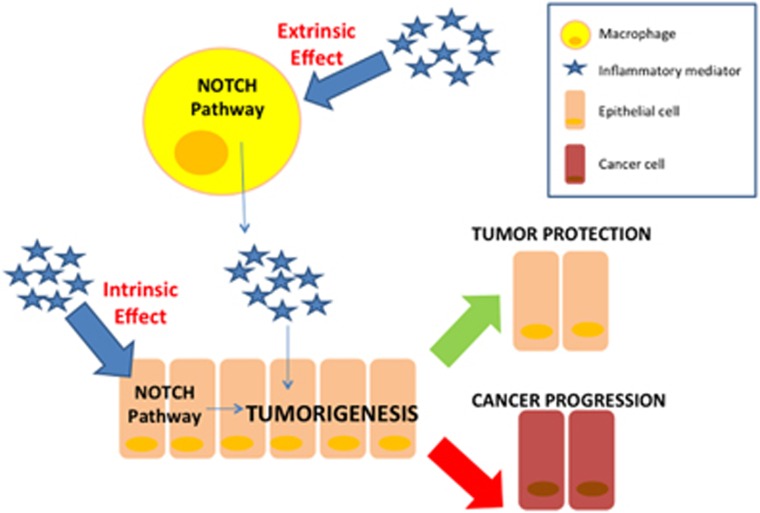
*Extrinsic and intrinsic effect of inflammatory-driven Notch activation on tumorigenesis*. When Notch signaling is activated in macrophages, it can induce the production of specific inflammatory mediators which in turn stimulate epithelial cells: thus, although not occurring into the epithelial cell, the dysregulation of the Notch pathway can indirectly exert a control on tumor progression (extrinsic effect). Alternately, the inflammatory milieu can directly modulate the Notch signaling within the epithelial cells, regulating several molecular processes involved in tumorigenesis (intrinsic effect)

Interestingly, inflammatory cells share and/or modulate some of the signaling molecules of the tumor cells, including those belonging to the Notch canonical and non-canonical signaling pathways.^9^ Thus, inflammation could have an ‘intrinsic' effect, specifically stimulating the Notch pathway in the epithelial cells; likewise, inflammation could have an ‘extrinsic' effect on tumor progression, since it modulates Notch within cells of the inflammatory compartment, that in turn are able to interact with tumor cells (Figure 1). The involvement of inflammatory mediators in the regulation of Notch signaling is documented in many malignancies, including breast cancer,^10^ multiple myeloma,^11^ hepatocellular carcinoma^12^ and colorectal cancer.^13, 14, 15^

Given these premises, an intriguing overview for understanding the ambiguity of Notch signaling in tumors relies on its crosstalk with the inflammatory *milieu*. Therefore, in this review we will examine the state of the art concerning the influence of inflammation on the biological effect of the Notch signaling in different types of cancer, with a particular focus on the intestinal epithelium.

## Canonical Notch pathway

The Notch pathway, which is able to regulate many different biological functions, relies on a cell-to-cell model of communication.^16^ In humans, the canonical Notch cascade begins when one of the specific trans-membrane Notch ligands of the sending cell (Jagged1-2, DLL1, DLL3 and DLL4) binds to one of the Notch receptors (Notch1–4) expressed on a receiving cell surface. The receptor-ligand binding triggers two consecutive proteolytic cleavages in the Notch receptor. The first proteolytic event, catalyzed by the TACE metalloproteinase (ADAM17), cleaves the extracellular portion of the receptor; the second proteolytic step involves the remaining membrane-anchored fragment, which is processed by the γ-secretase enzyme, and induces the release of the active intracellular domain of Notch (NICD). NICD translocates into the nucleus where it interacts with the transcriptional repressor protein CSL/RBP-J. Following the recruitment of Mastermind-like co-activators and the histone acetyltransferase p300, CSL/RBP-J is converted to a transcriptional activator leading to the induction of downstream target genes, including Hes1 and Notch-regulated ankyrin repeat protein 1.^17^

## Non-canonical Notch pathway

Importantly, to date a non-canonical role for Notch signaling has been reported, especially regarding the immune system. The non-canonical Notch pathways are RBP-Jκ-independent signals involved in several physiological and pathological cellular processes, including oncogenesis.^18^ A role for non-canonical Notch signaling in transformed cells has been suggested by the evidence that inhibition of *γ*-secretase does not block all Notch-related functions in tumor cells.^19^ The principal mechanisms able to interact in a non-canonical manner with Notch and involved in the response to inflammation are: I- the pathway of the nuclear factor kappa-light-chain-enhancer of activated B cells (NFkB);^20, 21^ II- hypoxia;^22, 23, 24, 25, 26, 27^ III- the epithelial-to-mesenchymal transition (EMT), in particular involving Transforming growth factor-beta (TGF-β),^28^ and matrix metalloproteinases (MMP9);^29^ IV- the Wnt signaling pathway, affecting the stability of β-catenin;^9, 30^ V- the mitogen-activated protein kinase (MAPK) and nutrient sensor kinase mTOR.^31^ Table 1 shows the principal molecular mechanisms concerning the inflammation-driven non-canonical Notch pathways in the field of malignant progression.

## The Notch pathway: oncogenic or tumor-suppressive role?

Although there has been extensive research on Notch deregulation in cancer in the last two decades, the biological effects upon Notch signaling activation are still not fully understood. Indeed, some reports clearly describe a tumorigenic activity of this pathway^32^ but, on the other hand, a tumor suppressor function of Notch signaling has also been reported.^33^

For instance, while studies demonstrated that the truncated form of Notch4 has a causative role in the development of mammary tumors in animal models,^34^ others reported a possible oncogenic role of Notch1 overexpression in human breast cancer tissues.^35^

Another context in which Notch exerts a tumor-promoting role is melanoma: indeed, global gene expression profiling revealed an overexpression of Notch receptors in primary human malignant melanomas.^36^ Importantly, it was demonstrated that the activation of Notch1 enables primary melanoma cells to gain metastatic capability via *β*-catenin.^37^

On the other hand, a protective role of Notch in other tumor settings has been reported. In a model of small cell lung cancer Sriuranpong and colleagues demonstrated that the overexpression of the active forms of Notch1 and Notch2 causes the block of cell cycle at G1 phase and the arrest of the tumor growth.^38^

Furthermore, Notch1-deficient animals spontaneously develop basal cell-carcinoma-like tumors associated with upregulation of Shh signaling. The authors also found that Notch1 deficiency leads to increased expression of *β*-catenin expression in the epidermis, which was reverted by the re-introduction of a dominant active form of the Notch1 receptor.^39^ Consistently with these findings, it has been reported a reduced expression of Notch1, Notch2 and Jagged1 in human basal cell carcinomas.^40^

Taken together, these data highlight that the activation of Notch pathway can trigger both oncogenic and tumor-suppressive functions depending on the specific cell and tissue context.

## Inflammation and cancer: the oncogenic role of Notch

The rationale for studying the inflammation-mediated carcinogenesis arises from the evidence that chronic inflammation is a known unfavorable condition, which predisposes to the onset of cancer; moreover, most solid tumors are characterized by an intrinsic tumor-promoting inflammatory response.^41^ For example, Rokavec and colleagues reported a feedback loop among Interleukin (IL)-6, STAT3 and miR34a, able to increase the invasiveness of colorectal cancer (CRC) cells.^42^ In a mouse model of colon cancer, the overexpression of IL-8 induces cancer growth and metastatization.^43^

Several studies demonstrated a strong correlation between Notch signaling and specific inflammatory mediators. It is known that high expression levels of Jagged1, Notch 1 and Notch2 correlate with tumor progression of myeloma;^44^ in this context, it has been recently proposed an activating role of Notch on IL-6 proliferating signals in the bone marrow niche, which results in an enhancement of tumor growth.^45^

In a mouse model of pancreatic cancer, it has been found that the crosstalk between TNF-*α*, the basal Notch signaling and Ikk2 (the Inhibitor of *κ*B kinase 2, a component of the NF-*κ*B signaling) induces the suppression of the nuclear receptor Pparg, which encodes for the anti-inflammatory nuclear receptor Ppar*γ*. In particular, the Hes1-mediated suppression of Pparg perpetuates the autocrine inflammatory activity of tumor pancreatic cells, inducing the production of inflammatory mediators, such as TNF- *α*, IL-6 and IL-1*β*. Therefore, through this loop, inflammation sustains the pancreatic cancer progression through the activation of the Notch pathway.^46^

A role for TNF-*α*/IKK*α* in the regulation of Notch1 signaling has also been reported in liver cancer cell lines: it has been proposed that the phosphorylation of FOXA2 (critical gene required for bile acid homeostasis), by IKK*α*, leads to activation of Notch1 signaling through downregulation of NUMB, thereby inducing tumorigenesis.^47^

Sansone *et al.* demonstrated that IL-6 is able to induce cancer stem cell renewal via Notch3 in an *in vitro* model of breast cancer.^48^ Another study showed that a gamma secretase inhibitor, able to block the Notch signaling and to attenuate the stem-like phenotype of cancer cells, reduced the T-cell-mediated production of both IL-6 and IL-8 in an *in vitro* model of inflammatory breast cancer.^10^ Another interesting interaction between the pro-inflammatory cytokine IL-1 and Notch1-4 has been reported in breast cancer, where Leptin, a well-defined pro-proliferation factor, is the link that leads to the expression of pro-angiogenic molecules, promoting cell proliferation and migration.^49^

In tongue squamous cell carcinomas, the IL-1*β* upregulates CXC chemokine receptor 4 (CXCR4), that mediates cancer growth and metastasis, leading to the concomitant activation of extracellular sregulated kinase (ERK); interestingly, the pharmacological inhibition of Notch1 signaling reversed this up-regulation.^50^

These multiple lines of evidence support the idea that pro-inflammatory *stimuli*, such as IL-1*β*, IL-6, IL-8 and TNF-*α* can lead to the activation of Notch signaling with a tumor-promoting effect on epithelial cells.

## Inflammation-mediated tumor suppressor role of Notch

As previously addressed, some data support a possible protective role of Notch signaling towards cancer progression^51^ and also in this case inflammation plays an important role. An example is the work of Talora and colleagues, in which they demonstrated that HPV-positive cervical carcinoma cell lines express significantly lower levels of the Notch1 indicating a protective role in infected keratinocytes.^52^

Another evidence is that NF-kB blockade and oncogenic Ras trigger invasive human epidermal neoplasia through TNF/JNK activity.^53^ Since Notch activation leads to induction of NF-κB,^54^ an attractive possibility is that the tumor suppressing function of Notch in keratinocytes is mediated by NF-*κ*B. Another context in which pro-inflammatory factors can drive a tumor-suppressive role for Notch is within the endothelium,^55^ where specific pro-inflammatory cytokines play a pivotal role in regulating functions of endothelial cells.^56^ Although this regulation is not directly connected to tumorigenesis, it is important to highlight that endothelial cell-fate is implicated in angiogenesis.^57^ An example is provided by Quillard *et al.*, who found that the inflammatory cytokines TNF-α and IL-1β lead to overexpression of Notch2 over Notch4, promoting apoptosis.^58^

On the other hand, other works support the hypothesis that proinflammatory factors, such as IL-6, may positively contribute to the abnormal angiogenesis in cancer.^59^

The previous examples support the hypothesis that, in some specific cases, the inflammation-dependent activation of Notch signaling could result in tumor-suppressive effects.

## Notch activation in intestinal inflammation

**Figure 2:**
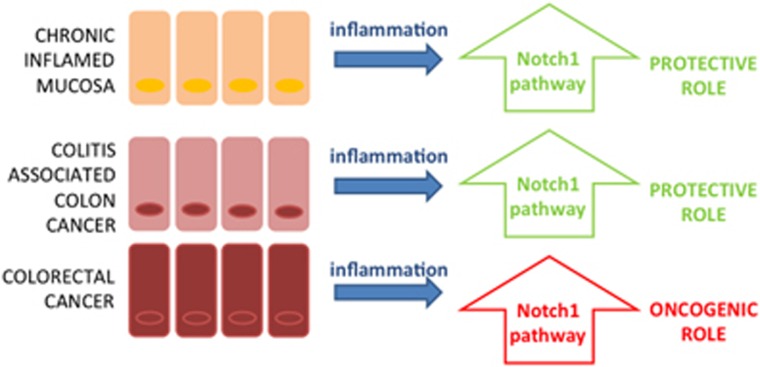
Notch1 function on tumorigenesis depending on the type of inflammatory stimulus on intestinal epithelia

The interplay between inflammation and Notch is particularly intriguing in the context of the intestinal epithelium (Figure 2).

In the intestinal mucosa, Notch signaling is crucial for the maintenance of the stem cell phenotype, as well as for determining cell-fate.^60^ In particular, the balanced composition of the four types of intestinal epithelial cells is essential for intestinal homeostasis as well as for host defense functions. ATOH1, repressed by the Notch target gene Hes1, is a master regulator for differentiation of secretory cell lineages.^61, 62^ In this scenario, Notch activation is necessary for epithelial regeneration after an inflammatory injury (such as in ulcerative colitis) where a depletion of secretory cells is observed.^15^ Interestingly, Kim and colleagues showed that the activation of Notch in the Apc^min/+^ mouse model converted intestinal high-grade into low-grade adenomas, suggesting a negative effect on cancer progression. They demonstrated that this mechanism is mediated by the negative control of Notch-regulated ankyrin repeat protein 1 on WNT target genes.^63^ The involvement of WNT/*β*-catenin in the protective role of Notch has also been demonstrated in an *in vivo* model of colitis-associated cancer (CAC), indicating that these pathways (Notch and WNT) cooperate even under sustained inflammation.^64^ More recently, an innovative link between the above mentioned protective role of Notch and inflammation has been proposed by Taniguchi and his group. They showed that gp130, a co-receptor for IL-6, triggers activation of Yes-associated protein (YAP) and Notch, independently of the classic gp130 effector STAT3, in order to stimulate epithelial cell proliferation and confer resistance to mucosal erosion.^65^

In the context of colorectal cancer and inflammation, a further mechanism that explains the role of Notch activation in carcinogenesis is related to Matrix metalloproteinases-9 (MMP9), a protein involved in the epithelial to mesenchymal transition (EMT). Garg and colleagues demonstrated that MMP9, which is a mediator of pro-inflammatory response, plays a protective role in the AOM/DSS mouse model of colitis-associate colorectal cancer, by activating p21WAF1/Cip1, which in turn modulates Notch1 and suppresses β-catenin.^66^ Intriguingly, in a different model of intestinal inflammation the role of MMP9 has also been related to an oncogenic function of Notch signaling. Indeed, Pope and his collaborators recently postulated that the up-regulation of Claudin-1, an integral component of the tight junctions structure, induces MMP9 and p-ERK signaling, leading to subsequent activation of Notch signaling, which in turn decreases goblet cell number thus enhancing susceptibility to mucosal inflammation.^67^ This evidence is in accordance with our recent *in vitro* work in which we demonstrated that the Notch1 pathway is activated in CRC cells in an MMP9-dependent manner under the *stimulus* of a complex mixture of pro-inflammatory factors obtained by activated macrophages.^29^ Indeed, other reports sustain the role of inflammatory factors in promoting Notch pathway activation and colon cancer progression, for example through the IL-6/Notch1/CD44 signaling axis.^68^ Table 2 summarizes the different roles of MMP9 realtive to the Notch activity.

While the common object is Notch signaling, what effectively changes among the above-mentioned reports is the ‘type' of inflammatory context, which profoundly differs in colitis, CAC or in the inflammatory microenvironment of CRC, as explained in the next section. Therefore nowadays, what we can affirm concerning the role of Notch activation and its complex interplay with inflammatory processes in intestinal epithelium is that the ‘quality' of the inflammation and the tissue-specific characteristics certainly influence the biological meaning of the activated pathway. Further studies are needed to increase our knowledge regarding the context specific function of Notch.

## Notch in immune system

In the previous sections we approached the issue of how the Notch signaling can be ‘bidirectionally' regulated by inflammatory context in epithelial or cancer cells. However, the modulation of Notch occurs in immune cells as well.^69^ Since the polarization of myeloid cells, primarily macrophages, can influence carcinogenesis, this topic has to be taken into account for a complete understanding of the relationship between Notch and inflammation in cancer progression.

Depending on environmental signals, macrophages can be differentially activated: they can be classically activated (M1 phenotype) or alternatively activated (M2 phenotype). While M1 macrophages are characterized by production of inflammatory mediators in response to microbial product-mediated activation of Toll-like receptors, M2 macrophages express less inflammatory molecules and play a key role in host defense and resolution of inflammation.^70^ Specific inflammatory mediators are expressed in relation to the context; for instance, during the transition from acute to chronic inflammation of colitis, a switch from Th1-Th17 derived cytokines to a prevalent Th2 inflammatory mediated response occurs.^71^

Several reports link Notch activation to macrophage functional phenotypes. Outz *et al.* demonstrated that Notch1 deficiency regulates vascular endothelial growth factor Receptor-1 (VEGFR-1) and inflammatory cytokine expression in macrophages, in particular Tumor Necrosis Factor-alpha (TNF-α), inducing a decrease of inflammation during wound healing.^72^ In particular, Notch1 system activation in macrophages drives the acquisition of the M1 phenotype, through the axis RBP-J-TLR4-IRF8.^73^ A recent study identified a novel function of Numb, a negative regulator of Notch1 signaling, in the induction of TNFα, IL-6, and IL-12 cytokine production in macrophages. Furthermore, Numb interacts with Itch that, in turn, regulates downstream signaling pathways, including NF-κ B p65 and p38 MAPK. Interestingly, the authors also report that sustained Notch activity in bone marrow, as a result of interrupting Numb, do not affect monocyte differentiation into macrophages, and speculate that Numb may influence cellular differentiation in a context-dependent manner.^74^

An appropriate example of the impact of Notch signaling on inflammatory responses is represented by cardiovascular disorders, such as myocardial infarction or atherosclerosis, and Leukemia, since Notch receptors and ligands are shared or simultaneously modulated by inflammatory effectors as well as endothelial cells.^55^ An example is provided by a mouse model of atherosclerosis and metabolic disorders resembling the cardiometabolic syndrome obtained by feeding LDL-receptor–deficient (*Ldlr*−dlrmple is provided by a mouse model of atherosclerosis and metabolic disorders resembling the cardiometabolic syndrome obtained by feeding LDL-receptor–deficient (sclerosis, and Leukemia, since Notch receptoy, reduces MCP-1 expression and attenuates the proinflammatory phenotype of macrophages, thus demonstrating that Notch signaling is able to drive proinflammatory programs of gene associated with the cardiometabolic syndrome. In particular, a central role seems to be played by DLL4, which acts both in homotypic and heterotypic crosstalk between different pathways that control inflammatory responses.^75^

Taken together, these data suggest that Notch, especially Notch1 and Notch3, appears to be a regulatory pathway controlling the balance of the immune system.

## Targeting inflammation to control the Notch pathway

Given its dichotomy between tumor-promoting and -suppressing function, and at the same time given its important implications in tissue homeostasis, direct intervention on Notch signaling as a target for cancer therapy is a delicate issue. When its precise function, in terms of positive or negative regulation of tumorigenesis, is clearly defined (at present only in specific *in vitro* or *in vivo* models), then the manipulation of Notch could be a relevant therapeutic target. This is the case of the employment of the γ-secretase inhibitor DBZ for the conversion of metaplastic Barrett's epithelium into post-mitotic goblet cells^76^ or in mouse models of familial adenomatous polyposis.^77^ However, since Hath1 mediates the effects of γ-secretase inhibitor, it has been proposed that only the subset of colorectal cancers that retain Hath1 expression could respond to the treatment.^78^ This evidence suggests that the pharmacological manipulation of the Notch pathway should be considered with caution and requires an in-depth knowledge of the related context.

Besides this approach, another attractive target for molecular intervention could be aimed at controlling the inflammatory processes which in turn modulate the Notch signaling. Notably, Chang Mo Moon and his group found that treatment with NSAIDs (indomethacin, sulindac and aspirin) has a suppressing effect on cancer stem cells both in an *in vitro* and in a xenograft model of colorectal cancer. Importantly, they contextually explored the modulation of Notch signaling, and they found that the effect of inhibition on colosphere formation is related to the downregulation of Notch/Hes1 signaling and to the upregulation of PPARG.^79^

Similarly, epidemiological evidence suggests that diet supplementation with anti-inflammatory agents exerts a protective role toward tumorigenesis.^80^ Noteworthy, our *in vitro* and *in vivo* studies revealed that the omega-3 polyunsatured fatty acids (ω-3 PUFAs), which are natural anti-inflammatory compounds, and in particular Eicosapentaenoic Acid is able to counteract the Notch pathway at normal expression levels in different settings of inflammatory-related colorectal cancers.^29, 66, 81^

## Conclusion

The crosstalk between inflammation and Notch signaling is extremely complex, due to the multifactorial nature of the inflammatory stimulus, which is context-specific, as well as for the duality of the Notch expression pattern. We analyzed how different effects of the Notch pathway in terms of biological meaning could be at least in part explained by the influence of the inflammatory context. We explored how this interaction generates a large number of cell type-specific responses.

In this scenario, the improvement of the knowledge regarding the molecular mechanisms at the basis of this interaction is indispensable to achieve adequate and innovative therapies.

## Figures and Tables

**Table 1:** Link between Pathways related to inflammation and non-canonical Notch pathways: involved molecular mechanisms

*Inflammation-linked pathway*	*Molecular mechanism*	*Effect*	*Model*	*References*
NFκB Signaling	Induction of Jagged 1 expression in non-cancer stem cells	Stimulation of Notch signaling in cancer stem cells	Basal-like breast cancer cell lines	^21^
	Induction of PI3K/Akt pathway	Activation of Notch1, tumor growth	Human melanoma samples and cell lines	^23^
Tissue Hypoxia	Stabilization of HIF-1a	Activation of Notch, tumor growth	Human melanoma samples and cell lines	^23^
	Stabilization of Hif-1a	Augmented Notch1 signaling, altered expression of cell cycle regulatory proteins, accelerated cell proliferation	T-cell acute lymphoblastic leukemia cells	^24^
	Induction of Notch pathway, up-regulation of Notch ligand expression	Induced EMT, E-Cadherin down-regulation, expression of Snail1	Cell lines of cervical, colon, glioma and ovarian cancer; breast cancer	^26, 27^
	Induction of 66-kDA isoform of the SHC gene (p66Shc)	Induction of Notch3 signaling, self-renewal (induction of Jagged1) and hypoxia survival	Mammospheres	^25^
Epithelial to mesenchymal transition (EMT)	Transforming growth factor-beta (TGF-b) induction	Expression of Hey1 and Jagged1	Epithelial cells from mammary gland, kidney tubules and epidermis	^28^
WNT	*β*-catenin/TCF-mediated transcriptional activation of Jagged1	Activated Notch1 and Notch2 in tumors containing nuclear *β*-catenin	Colorectal cancer cells, human tumors from FAP	^30^

**Table 2:** Role of interaction between MMP9 and Notch pathway in colon carcinogenesis

*MMP9 function*	*Molecular mechanism*	*Effect on Notch pathway*	*Model*	*References*
Protective role against CAC	Activation of p21WAF1/Cip1, suppression of b-catenin	Increased Notch1 activation	MMP9-/- and WT mice; AOM/DSS mouse model	^66^
Enhanced susceptibility to mucosal inflammation	Claudin-1 induced activation	Activation of Notch signaling, inhibition of differentiation of intestinal epithelial cells into goblet cells, decrease of Muc-2 positive cells	Villin-claudin1 transgenic mouse model	^67^
Oncogenic role in CRC	Inflammation-driven activation	Overexpression of NICD and Jagged1; induction of Notch-regulated ankyrin repeat protein 1	*In vitro* model of interaction between macrophages and CRC cells	^29^
